# The habit of adding salt to food at the table and its association with socio-demographic, anthropometric and dietary characteristics in Brazilian older adults

**DOI:** 10.3389/fpubh.2026.1737516

**Published:** 2026-04-02

**Authors:** Flávia dos Santos Barbosa Brito, Ariane Cristina Thoaldo Romeiro, Débora Martins dos Santos, Carla Gonçalves, Maria Eduarda Sant'Anna, Alexandre dos Santos Brito, Amanda Rodrigues Amorim Adegboye

**Affiliations:** 1 Instituto de Nutrição, Departamento de Nutrição Social, Universidade do Estado do Rio de Janeiro, Rio de Janeiro, Brazil; 2 Centro Universitário Serra dos Órgãos, Teresópolis, Brazil; 3 CITAB—Centre for the Research and Technology of Agro-Environmental and Biological Sciences, University of Trás-os-Montes and Alto Douro, Vila Real, Portugal; 4 Instituto de Estudos em Saúde Coletiva, Universidade Federal do Rio de Janeiro, Rio de Janeiro, Brazil; 5 Centre for Agroecology, Water and Resilience (CAWR), Coventry University, Coventry, United Kingdom; 6 Centre for Healthcare and Communities (CHC), Coventry University, Coventry, United Kingdom

**Keywords:** dietary behavior, gender difference, older adults, salt, socio-demographic factors, sodium

## Abstract

**Introduction:**

Adding salt to food at the table is a recognized behavior linked to long-term preference for salty foods and overall daily salt intake. Given its impact on quality of life and life expectancy, several consumer awareness campaigns target the reduction of discretionary salt use. To inform such strategies in aging populations, we aimed to explore the association between socio-demographic, anthropometric and dietary characteristics and behavior regarding adding salt to food at the table among Brazilian older adults.

**Methods:**

This was a cross-sectional study that analyzed food consumption data from 8,336 Brazilians aged ≥ 60 years who participated in the 2017–2018 National Dietary Survey of the Household Budget Survey.

**Results:**

The results indicate that the prevalence of adding salt to food at the table was significantly higher among men than women (12.7% vs. 9.4%, *p* < 0.001). Among men, two factors were significantly associated with adding salt to food at the table. Men who reported not following a diet for high blood pressure were more than twice as likely to add salt compared with those on such a diet (adjusted OR = 2.44, 95% CI: 1.13–4.54). In addition, men living alone had a 62% higher likelihood of adding salt compared with those living with others (adjusted OR = 1.62, 95% CI: 1.08–2.43). Among women, the odds of adding salt to food at the table was 68% higher among those not following a diet for high blood pressure (adjusted OR = 1.68, 95% CI: 1.09–2.60), 81% higher among those who did not consume fruits (adjusted OR = 1.81, 95% CI: 1.33–2.47), and 40% higher for those not consuming vegetables (adjusted OR = 1.40, 95% CI: 1.00 – 1.97), and more than twice as high among those have high contribution of ultra-processed foods in the diet and those living in urban areas.

**Conclusion:**

The habit of adding salt to food at the table differs between men and women and is influenced by sociodemographic and dietary factors, particularly among women. Public health policies in Brazil should take these gender differences into account to promote healthier eating habits and reduce the burden of chronic noncommunicable diseases among older adults.

## 1 Introduction

Salt, also known as sodium chloride, is one of the oldest seasonings used in cooking (1). Salt has always played an important role as a flavoring and food preservative, but due to changing lifestyles and the proliferation of processed and ultra-processed foods in recent decades, the amount of dietary salt consumed has become a public health problem (2–6). Data from the Global Burden of Disease (GBD) study showed that, in 2021, approximately 1.86 million deaths and 41.3 million Disability-Adjusted Life Years (DALYs) were attributable to high salt intake, reflecting the most recent global burden estimates available (7, 8).

Excessive salt intake is associated with several adverse health outcomes (9–12) and can particularly affect older adults (13–15). High salt intake can accelerate cognitive decline and neurodegenerative diseases and can affect an individual's stability and mobility, further exacerbating the existing challenges faced by many older adults (13, 16).

Data from various countries show that most populations consume significantly more salt than the World Health Organization's (WHO) recommended limit of 5 grams per day (g/d) for adults (WHO, 2013). Globally, the average salt intake for adults is estimated at 10.78 g/d (70) more than double the recommended amount. In Brazil, the median sodium intake among adults was estimated at 2,432 mg/day (equivalent to 6.1 g of salt) based on the 2017–2018 National Dietary Survey (NDS), exceeding the WHO limit but notably lower than the 9.34 g/day estimated in 2013 (17, 18).

Although most of the salt comes from ultra-processed foods (2), discretionary salt use, including adding salt to food at the table, remains a significant contributor, accounting for 6–20% of total salt intake (19, 20). According to data from the 2017–2018 Household Budget Survey (HBS), the habit of adding salt to food at the table was reported by 13.5% of the population (21). However, no detailed information on the prevalence of this behavior across different age groups or its associated factors has been reported (21).

The habit of adding salt to food at the table, is a good indicator of a person's preference for salty foods and is directly related to socio-demographic characteristics such as sex, age and socioeconomic status (14, 22–24). Effective salt reduction policies must integrate strategies that address both discretionary salt use, such as adding salt to food at the table and the reduction of salt content in processed foods. Therefore, assessing the habit of adding salt to food at the table and identifying the characteristics associated with this behavior is crucial. Such information can help raise awareness among older adults of the need to change this habit and, consequently, contribute to public health policies designed to target specific population groups with salt education campaigns.

In Brazil, limited research exists on the socio-demographic, anthropometric or dietary characteristics associated with the habit of adding salt to food at the table among older adults. Therefore, this study aimed to investigate the association between socio-demographic, anthropometric and dietary characteristics and the habit of adding salt to food at the table, contributing to a better understanding of this behavior that can potentially inform salt reduction interventions.

## 2 Materials and methods

### 2.1 Survey description

The 2017–2018 Household Budget Survey (HBS) is the sixth conducted by the Brazilian Institute of Geography and Statistics (Instituto Brasileiro de Geografia e Estatística - IBGE, in Portuguese), which assesses the structures of consumption, expenditures, income and part of the asset variation of the households, providing a profile of the life conditions of the population based on the analysis of the household budgets (25). IBGE is the official Brazilian Population Statistics Agency and the main provider of data and information about the country. The last two HBSs included the Brazilian National Dietary Survey (NDS), which assessed individual food consumption in a sub-sample of the households surveyed in the HBS, with data from all individuals aged 10 and over living in the households. Nutrition specialists from the Ministry of Health provided technical expertise to the study design, data validation, and publication process (21).

This study used secondary data form the surveys which are all publicly and freely available. The microdata, which includes data, documentation, questionnaires, table translators, reading software and calculation memory, is freely available on the IBGE website: https://www.ibge.gov.br/en/statistics/experimental-investigations/experimental-statistics/25610-pof-2017-2018-pof-en.html?edicao=28652&t=microdados (accessed October 17, 2025). Microdata ensures confidentiality by omitting identifiable information, such as household addresses, telephone numbers and census tract numbers. Brazilian census data are protected by law (Law No. 13,709/2018 — General Data Protection Law). Supplementary Law No. 105/2001, which ensures that confidential information is not made available to the public. The IBGE the 2017-2018 HBS and NDS surveys following the ethical Declaration of Helsinki.

### 2.2 Study design and population

This is a cross-sectional study using data from the Brazilian NDS and the HBS, both conducted by the IBGE in 2017–2018. The NDS was conducted simultaneously in a random sub-sample of 34.7% of the HBS. The HBS used the IBGE Integrated Household Survey System, which selects a “Master Sample,” defined as a set of census tracts that cover the entire national territory. The complex sample was selected in two stages. The Primary Sampling Units (PSUs) were derived from the master sample, which consists of a set of census sectors. The PSUs were selected by sampling with probability proportional to the number of households in each sector. The census sectors were previously obtained by government administrative divisions, urban or rural settings and income levels based on the Demographic Census of the year 2010. In the second stage, the households were selected by simple random sampling. The sectors were distributed over the four quarters of the year in which the research was carried out. For the assessment of individual food consumption, a sub-sample of households was selected from the sample by simple random sampling.

**Figure 1:**
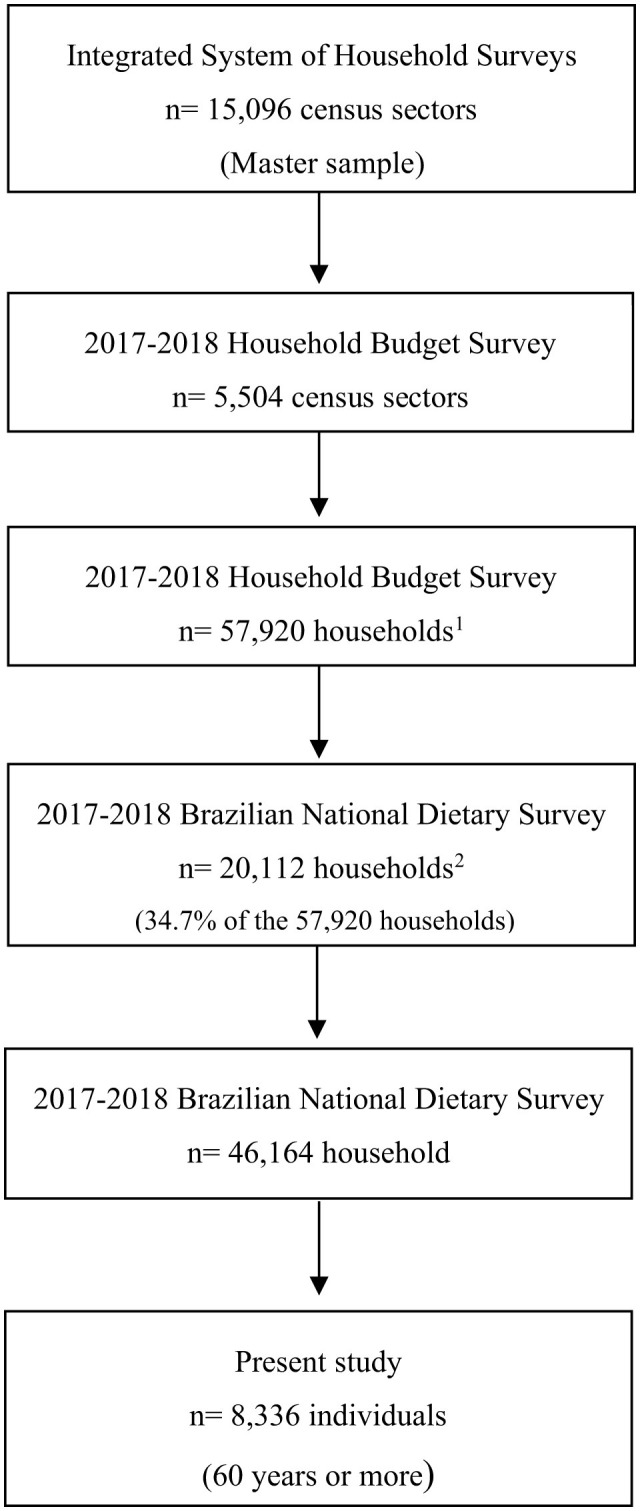
Sample flow chart in 2017–2018 Household Budget Survey and Brazilian National Dietary Survey. ^1^Households randomly selected from the predefined stratification system. An average loss of 15% was estimated due to possible refusals to answer the survey, and the same proportion was added to the final number of households to minimize possible losses. ^2^Households randomly selected from the predefined stratification system.

The 2017-2018 HBS, consisted of 5,504 selected sectors and 57,920 investigated households, with 20,112 households randomly selected from the 57,920 households surveyed in the HBS. All individuals aged 10 and over in the households surveyed in the 2017–2018 HBS were invited to participate in the NDS (21), resulting in a sample of 46,164 individuals aged 10 years or older. The final sample for this study included 8,336 individuals aged ≥ 60 years (Figure 1). Additional information on the sampling process can be obtained from official publications of the IBGE (21, 25).

### 2.3 Data collection

Data collection was carried out over 12 months, from July 2017 to July 2018. The information collected in the 2017–2018 HBS was based on seven modules. For this study, only modules 1 and 7 were used: the former assessed sociodemographic characteristics, and the latter assessed individual food consumption. Further information about data collection can be found in a previous publication by IBGE (21, 25).

### 2.4 Data collection

Individual food consumption data were collected using two 24-h dietary recalls on non-consecutive days during the week when a trained IBGE agent was present in the household. This collection process was uniform across the entire sample to ensure representativeness in each quarter of the year and to account for seasonal variations in food consumption (21).

Both 24-h dietary recalls periods were collected through face-to-face interviews, with an 84% response rate for both periods. These 24-h dietary recalls were collected using the USDA Automated Multiple-Pass Method (AMPM), which is a structured, computer-assisted, multiple-pass method developed by the US Department of Agriculture (USDA). The AMPM has five steps. Each step guides the respondent through the recall process, prompting them to provide more detailed information and reducing the likelihood of omissions (26).

This process was carried out using software developed specifically for this assessment and included in the tablet used by the interviewer. Food quantities consumed (in grams) were estimated using the reference table of food portion sizes in Brazil, originally developed in the 2008–2009 NDS-HBS and updated in the 2017–2018 edition (27).

Out-of-home food consumption included all food and beverages purchased outside the home and consumed without going through the household supply. For the present study, out-of-home food consumers were defined as individuals who reported that they had consumed at least one item of food away from home.

The choice of 24-h dietary recalls was based on the fact that it is almost universally used in population-based research and is considered the method with the lowest possibility of systematic error. Another reason was the results of the instrument validation study conducted in the 2008–2009 NDS, which showed better performance of 24-h dietary recalls than food records using double-labeled water as the gold standard method. Furthermore, IBGE pretested and validated the collection instruments, performed quality control procedures during data collection and deleted inconsistent records and replaced them with imputed values in order to minimize the biases inherent in the use of dietary surveys (21, 28).

### 2.5 Usual dietary intake

The Multiple Source Method (MSM) was used to estimate the usual intake of food in general. MSM is an open-access statistical program developed by the European Prospective Investigation into Cancer and Nutrition (EPIC) (29, 30) and is available online at https://nugo.dife.de/msm/ (accessed October 17, 2025). This method eliminates within-person variance in consumption using a three-part mixed model, which requires at least two days of short-term dietary assessments (e.g., 24-h dietary recalls) (31). In the first step, the probability of eating a particular food on a random day is estimated for each individual. Second, the usual amount of food consumed in a day is estimated. Finally, the resulting numbers from steps one and two are multiplied by each other to estimate the usual daily intake for each individual. This analysis used data from the two days of food consumption. A more detailed description of the method can be found in Haubrock et al. (29).

### 2.6 Classification of ultra-processed foods (UPF)

The Nova food classification system, developed by a team at the University of São Paulo, in Brazil, classifies all foods and food products into four groups according to the nature, extent and purpose of the industrial processing they undergo. It considers all physical, biological and chemical methods used during the food manufacturing process, including the use of additives (32, 33). Definitions and lists of examples for each of the four Nova groups can be found at https://www.fsp.usp.br/nupens/en/food-classification-nova/ (accessed October 17, 2025) and in Romeiro et al. (34).

All foods and beverages reported were classified as ultra-processed (UPF) or non-ultra-processed according to the NOVA food classification (33). All UPF were further divided into nine subgroups (1) sweet biscuits, cakes, and pies; (2) packaged salty snacks; (3) bread; (4) confectionaries; (5) soft drinks and refreshments; (6) milk-based drinks; (7) pizzas, hamburgers, sandwiches, and savories; (8) frozen or instant ready-made dishes; reconstituted meat products; and (9) others.

All food groupings were based not only on the Nova classification system, but also on the food composition, the list of ingredients, nutritional information, resolutions on norms and standards, food processing technology, and published studies (35–37). This process was carried out by two independent researchers. When discrepancies in classification occurred, they discussed these differences until reaching a consensus. In these cases, they resolved the issues by adopting the most conservative classification, i.e., the lowest degree of processing. However, there were some exceptions to this approach, including items such as bread, ready-to-eat cereals and salty snacks.

The high contribution of UPF in the diet of older adults was assessed by calculating the percentage contribution to the total energy of the diet (% of total energy). This percentage was categorized as less than the 75th percentile and greater than or equal to the 75th percentile, and then categorized as 0 = no days, 1 = one day and 2 = two days.

### 2.7 Dependent variable

Information on adding salt to food at the table was obtained from the following question in the Module 7 of the 2017–2018 HBS: “Do you have the habit of adding salt to food at the table?” with the answer alternatives (yes or no) (25). This was the main dependent variable.

### 2.8 Independent variables: socio-demographic, anthropometric and dietary characteristics of the older adults

The sociodemographic characteristics considered in this study were sex (male and female), age groups (10–19 years; 20–59 years and 60 years and over), years of education (0–4 years, 5–8 years, 9–11 years and 12 years and above), household arrangements (single person: household composed of a single person; couple: household composed of the head of household and spouse without children; mixed: other types of households composed in ways different from those above); household per capita income (in quartiles), Brazilian regions (North, Northeast, Southeast, South, Midwest); area of residence (urban, rural); currently being on a diet for high blood pressure (yes or no); eating out at least one day (yes or no); high contribution of UPF in the diet (0 = no days, 1 = one day, and 2 = two days); consumption of fruits (yes or no); and consumption of vegetables (yes or no). This last variable was constructed by selecting all vegetables consumed (excluding starchy vegetables such as potatoes, cassava, manioc, and yams).

Height and weight were self-reported. Weight status classification was based on body mass index (BMI). BMI was calculated as weight in kilograms divided by the square of the height in meters (kg/m^2^) and then classified according to Lipschitz (72), with the following cutoffs applied according to older adults (underweight (BMI < 22 kg/m^2^), normal weight (22 ≥ BMI < 27 kg/m^2^), overweight (BMI ≥ 27 kg/m^2^). The conversion of the Brazilian currency to US dollars was conducted based on the exchange rate as of 31 January 2018 (38). Per capita income was then divided into quartiles including a per capita monthly family income of less than US$ 226 (1st quarter), from US$ 226 to less than US$ 391 (2nd 414 quarter), from US$ 391 to less than US$ 678 (3rd quarter) and equal to or greater than US$ 678 (4th quarter). The variable “household arrangement” was constructed based on the residents' relationship, that is, their degree of kinship or nature of the existing subordination with the reference person (who was responsible for the household). The spouse was the resident who lived conjointly with the reference person. The child/children were those considered as legitimate, adopted or brought up by the reference person and/or spouse. The arrangement ‘single parent with children' was composed of the reference person in the household of both sexes and with at least one child. Mixed households were composed of other members with or without any degree of kinship with the reference person or spouse (e.g., son-in-law, daughter-in-law, parents, grandparents, grandchildren, siblings, domestic workers and other relatives).

### 2.9 Statistical analyses

All analyses were performed using Stata (StataCorp LP, College Station, version 16 TX, USA), stratified by sex, taking into account the complex survey design and the sampling weights applied using the *svy* command with the *subpop* option to specify the subpopulation of interest (31). Categorical variables were presented as relative frequencies (%) and their respective 95% confidence interval (CI). These proportions were compared using Pearson's chi-square test, corrected for the sampling design using Rao-Scott. The prevalence of the habit of adding salt to foods at the table according to characteristics of older adults by sex were presented as relative frequencies with their respective confidence intervals. Non-overlapping of confidence intervals was considered to identify the differences among sexes (39). Crude and multiple logistic regression were used to assess the association between the characteristics of the older adults and the addition of salt to foods at the table. Predictor variables with a *P*-value (*p*) < 0.20 in the crude analysis were included in the multiple logistic regression. Variables that remained associated (*p* ≤ 0.05) after adjustment for all other variables included in the analysis were retained in the model. These models allowed the calculation of the odds ratios (OR) and 95% CIs. Goodness of fit of logistic regression models was tested by using the “*svylogitgof* ” command in Stata which takes into account weights and sampling strategy (40). For all analyses, 2-sided *p* ≤ 0.05 indicated statistical significance.

## 3 Results

Of the 8,336 older adult participants, 55.9% (95% CI 54.6%−57.1%) were women. The characteristics of the study participants by sex are shown in Table 1. Men were more likely than women to add salt to foods at the table (12.7% vs. 9.4%, *p* < 0.001). In contrast, women were more likely to consume vegetables, fruit, have a higher contribution of UPF in their diet, report being on a diet for high blood pressure, and live in urban areas (*p* < 0.001). There were no significant differences between men and women in age group, years of schooling, per capita income and regions of the country (*p* > 0.001).

**Table 1:** Sociodemographic characteristics of the study population by sex. National Dietary Survey, Brazil, 2017–2018.

**Characteristics**	**Total (*****n*** = **8,336)**				**Male (*****n*** = **3,789)**				**Female (*****n*** = **4,547)**				***P*-value^a^**
	***n***	**%**	**95% CI** ^a^		***n***	**%**	**95% CI** ^a^		***n***	**%**	**95% CI** ^a^		
**Age groups**			
60-69	5,006	60.1	58.3	61.8	2,315	62.0	59.6	64.3	2,691	58.6	56.4	60.8	0.09
70-79	2,277	27.3	25.8	28.8	1,024	26.3	24.3	28.4	1,253	28.0	26.0	30.1	
80 and older	1,053	12.7	11.6	13.8	450	11.7	10.3	13.4	603	13.4	11.9	15.0	
**Education, years**			
0-4	3,929	38.6	36.9	40.4	1,834	39.0	36.6	41.2	2,095	38.4	36.2	40.7	0.49
5-8	2,295	28.9	27.2	30.6	1,044	29.5	27.3	31.8	1,251	28.3	26.3	30.5	
9 or more	2,112	32.5	30.6	34.5	911	31.6	29.2	34.2	1,201	33.2	31.0	35.5	
**Body mass index** ^b^			
Underweight	1,48	16.8	15.6	18.1	603	14.7	13.2	16.4	877	18.4	16.7	20.2	0.01
Normal weight	3,809	44.5	42.9	46.1	1,849	47.1	44.8	49.3	1,960	42.5	40.2	44.8	
Overweight	3,047	38.7	37.1	40.3	1,337	38.2	36.0	40.5	1,710	39.1	37.0	41.3	
**Per capita income, in quartile** ^c^			
1st	947	8.8	8.0	9.7	466	9.5	8.4	10.7	481	8.3	7.3	9.3	0.22
2nd	2,15	22.6	21.0	24.4	1,023	23.2	21.3	25.2	1,127	22.2	20.2	24.4	
3rd	2,624	30.6	28.7	32.5	1,174	29.8	27.6	32.1	1,45	31.1	29.0	33.4	
4th	2,615	38.0	36.0	40.0	1,126	37.6	35.1	40.1	1,489	38.4	36.1	40.7	
**Brazilian regions**			
North	849	5.5	4.9	6.14	418	9.5	5.1	6.7	431	5.3	4.4	6.2	0.54
Northeast	2,839	25.1	24.0	26.2	1,241	24.7	23.3	26.5	1,598	25.2	23.8	26.7	
Southeast	2,387	47.3	45.8	48.8	1,069	46.5	44.3	48.7	1,318	47.9	46.1	49.8	
South	1,309	15.7	14.8	16.6	589	16.0	14.7	17.4	720	15.4	14.4	16.5	
Midwest	952	6.5	5.9	7.10	472	6.8	6.0	7.7	480	6.2	5.5	6.7	
**Area of residence**			
Urban	6,324	86.1	85.2	86.9	2,689	83.0	81.7	84.2	3,635	88.6	87.6	89.4	0.01
Rural	2,012	13.9	13.1	14.8	1,100	17.0	15.8	18.3	912	11.5	10.6	12.4	
	***n***	**%**	**95% CI** ^a^		***n***	**%**	**95% CI** ^a^		***n***	**%**	**95% CI** ^a^		
**Household arrangements** ^d^			
Single person	1,356	15.9	14.7	17.2	551	13.9	12.3	15.6	805	17.6	15.9	19.4	0.01
Couple	2,391	28.7	26.9	30.6	1,313	35.4	33.2	37.7	1,078	23.4	21.6	25.2	
Mixed	4,589	55.4	53.4	57.4	1,925	50.7	48.3	53.1	2,664	59.1	56.8	61.4	
**Eating out at least one day**			
Yes	1,731	21.5	20.1	23.0	904	24.3	22.3	26.5	827	19.2	17.5	21.1	0.01
No	6,605	78.5	77.1	80.0	2,885	75.7	73.6	77.7	3,72	80.8	78.9	82.5	
**Diet for high blood pressure**			
Yes	1,347	14.6	13.4	15.8	479	11.5	10.1	13.2	868	16.9	15.4	18.7	0.01
No	6,989	85.5	84.2	86.6	3,310	88.5	86.9	89.9	3,679	83.0	81.4	84.6	
**Adding salt to food (at the table)**			
Yes	865	10.9	9.78	12.1	468	12.7	11.1	14.5	397	9.4	8.1	10.9	0.01
No	7,471	89.1	87.9	90.2	3,321	87.3	85.6	88.9	4,15	90.6	89.1	91.9	
**Consumption of vegetables**			
Yes	3,527	63.5	61.8	65.1	2,072	60.4	58.1	62.7	2,737	65.9	63.9	67.9	0.01
No	4,809	36.5	34.9	38.2	1,717	39.6	37.4	41.10	1,81	34.1	32.1	36.1	
**Consumption of fruits**			
Yes	4,182	47.8	45.9	49.8	1,634	45.5	42.8	48.1	2,548	57.5	55.2	59.8	0.01
No	4,154	52.2	50.2	54.1	2,155	54.6	51.9	57.2	1,999	42.5	40.2	44.9	
**High contribution of UPF in the diet**			
No day	6,670	76.2	74.6	77.8	3,134	78.5	76.2	80.6	3,536	74.4	72.4	76.3	0.01
One day	1,366	19.3	17.9	20.7	550	17.7	15.8	19.9	816	20.5	18.7	22.4	
Two days	300	4.5	3.0	5.3	105	3.8	3.0	4.9	195	5.1	4.2	6.2	

UPF, Ultra-processed food; CI, Confidence interval. ^a^Chi-square test with correction of Rao-Scott; P-value less than 0.05 is considered significant. ^b^Underweight (BMI < 22 kg/m^2^), normal weight (22 kg/m^2^ ≥ BMI < 27 kg/m^2^), and overweight (BMI ≥ 27 kg/m^2^). ^c^1st: < US$ 226; 2nd: ≥ US$ 226 to < US$ 391; 3r: ≥ US$ 391 to < US$ 678; 4th: ≥ US$ 678. ^d^Single person (household composed of a single person), couple (household composed of the head of household and spouse - without child), Mixed (other types of households composed in other ways than those above).

Table 2 shows the prevalence of adding salt to food at the table according to the characteristics of older adults, stratified by sex. Overall, this practice was more prevalent among men than women across sociodemographic, anthropometric, and dietary characteristics. Participants who were following a diet for hypertension reported markedly lower prevalence rates (6.1% of men and 5.6% of women) than those who were not (13.6% of men and 10.2% of women) (Table 2).

**Table 2:** Prevalence (%) of the habit of adding salt to foods at the table according to characteristics of older adults by sex. National Dietary Survey, Brazil, 2017–2018 (*n* = 8,336).

**Characteristics**	**Male (*****n*** = **3,789)**			**Female (*****n*** = **4,547)**		
	**%**	**95% CI**		**%**	**95% CI**	
**Age groups**			
60–69	13.3	11.2	15.7	10.8	8.9	12.9
70–79	12.1	9.5	15.3	8.1	6.2	10.6
80 and older	10.8	7.5	15.1	6.3	4.3	9.2
**Education, years**			
0–4	12.3	10.3	14.7	7.9	6.4	9.8
5–8	12.4	9.9	15.4	8.5	6.5	11.1
9 or more	13.4	10.1	17.4	11.9	9.3	15.2
**Body mass index** ^a^			
Underweight	11.0	8.2	14.6	8.2	6.0	11.1
Normal weight	14.2	11.8	17.1	9.3	7.3	11.9
Overweight	11.4	9.2	14.2	10.1	8.2	12.5
**Per capita income. in quartile** ^b^			
1^st^	15.8	12.1	20.3	9.0	6.3	12.7
2^nd^	12.5	10.0	15.5	8.5	6.3	11.3
3^rd^	10.7	8.6	13.3	10.3	7.7	13.7
4^th^						
**Brazilian regions**			
North	19.2	13.4	26.6	11.7	5.3	23.7
Northeast	10.8	8.9	13.0	6.9	5.4	8.7
Southeast	12.6	9.9	16.0	11.2	9.0	13.8
South	12.7	9.8	16.3	7.4	5.3	10.2
Midwest	14.6	10.3	20.4	9.4	6.1	14.2
**Area of residence**			
Urban	13.0	11.1	15.1	10.1	8.7	11.8
Rural	11.3	9.3	13.6	4.3	3.0	6.0
**Household arrangements** ^c^			
Single person	18.0	13.4	23.8	11.0	8.1	14.6
Couple	11.5	9.3	14.2	6.2	4.7	8.2
Mixed	12.0	9.9	14.5	10.3	8.4	12.5
**Eating out at least one day**			
Yes	12.3	10.5	14.2	9.3	7.9	11.0
No	14.0	10.8	18.0	9.9	7.2	13.5
**Diet for high blood pressure**			
Yes	6.1	3.4	10.6	5.6	3.8	8.1
No	13.5	11.8	15.5	10.2	8.7	11.9
**Consumption of vegetables**			
Yes	13.9	11.4	16.9	11.3	8.8	14.5
No	11.9	9.9	14.1	8.4	7.1	10.1
**Consumption of fruits**			
Yes	13.8	11.7	16.3	12.4	10.0	15.2
No	11.3	9.3	13.8	7.3	6.0	8.8
	**%**	**95% CI**		**%**	**95% CI**	
**High contribution of UPF in the diet**			
No day	12.7	11.1	14.6	8.3	6.9	9.8
One day	12.3	8.3	17.8	11.9	8.6	16.2
Two days	13.4	6.8	24.5	16.7	10.4	25.9

UPF, Ultra-processed food; CI, Confidence interval; P-value less than 0.05 is considered significant. ^a^Underweight (BMI < 22 kg/m^2^), normal weight (22 kg/m^2^ ≥ BMI < 27 kg/m^2^), and overweight (BMI ≥ 27 kg/m^2^). ^b.^1st: < US$ 226; 2nd: ≥ US$ 226 to < US$ 391; 3r: ≥ US$ 391 to < US$ 678; 4th: ≥ US$ 678. ^c.^Single person (household composed of a single person), couple (household composed of the head of household and spouse - without child), Mixed (other types of households composed in other ways than those above).

The results of the crude and multiple ratios of the associations stratified by sex are shown in Table 3. All variables with *p*-values less than 0.20 were selected for inclusion in the multiple analysis. Among men, we found that diet for hypertension and living alone were associated with adding salt to the table. Among women, age, household arrangement, area of residence, diet for hypertension, vegetable consumption, fruit consumption and a high proportion of ultra-processed foods in the diet were associated with adding salt at the table.

**Table 3:** Crude and adjusted linear regression of adding salt to food at the table with socio-demographic factors among older adults by sex. National Dietary Survey, Brazil, 2017-2018, (*n* = 8,336).

**Characteristics**	**Male (*****n*** = **3,676)**								**Female (*****n*** = **4,660)**							
	**Crude OR**	**95%CI**		***P*** **-value**	**adjusted OR**	**95% CI**		***P*** **-value**	**Crude OR**	**95%CI**		***P*** **-value**	**adjusted OR**	**95% CI**		***P*** **-value**
**Age group**			
60–69	1.27	0.82	1.97	0.28					1.79	1.14	2.80	0.01	2.02	1.25	3.26	0.04
70–79	1.14	0.71	1.84	0.58					1.31	0.79	2.16	0.29	1.47	0.87	2.46	0.15
80 and older	1								1				1			
**Education, years**			
0–4	1								1							
5–8	1.00	0.73	1.38	0.98					1.08	0.74	1.57	0.69				
9 or more	1.10	0.75	1.59	0.63					1.57	1.09	2.26	0.01				
**Body mass index** ^a^			
Underweight	1								1							
Normal weight	1.34	0.91	1.97	0.14					1.15	0.75	1.77	0.51				
Overweight	1.04	0.69	1.57	0.84					1.26	0.84	1.89	0.27				
**Per capita income, in quartile** ^b^			
1st	1.20	0.79	1.82	0.40					0.96	0.61	1.50	0.86				
2nd	0.92	0.63	1.34	0.65					0.90	0.60	1.34	0.60				
3rd	0.77	0.53	1.13	0.18					1.11	0.74	1.66	0.61				
4th	1								1							
**Brazilian regions**			
North	1.38	0.77	2.47	0.28					1.28	0.48	3.39	0.62				
Northeast	0.70	0.45	1.11	0.13					0.72	0.42	1.22	0.22				
Southeast	0.84	0.52	1.36	0.48					1.22	0.72	2.07	0.46				
South	0.85	0.52	1.39	0.51					0.78	0.43	1.39	0.39				
Midwest	1								1							
**Area of residence**			
Urban	1.18	0.89	1.55	0.25					2.52	1.68	3.76	0.01	2.44	1.60	3.72	0.01
Rural	1								1				1			
	**Crude OR**	**95%CI**		***P*** **-value**	**adjusted OR**	**95% CI**		***P*** **-value**	**Crude OR**	**95%CI**		***P*** **-value**	**adjusted OR**	**95% CI**		***P*** **-value**
**Household arrangements** ^c^			
Single person	1.61	1.07	2.41	0.02	1.62	1.08	2.43	0.02	1.08	0.73	1.60	0.71	1.14	0.76	1.70	0.53
Couple	0.95	0.69	1.31	0.76	0.95	0.69	1.31	0.77	0.58	0.40	0.84	0.01	0.58	0.40	0.85	0.01
Mixed	1				1				1							
**Eating out at least one day**			
Yes	1.18	0.83	1.64	0.38					1.07	0.72	1.60	0.73				
No	1								1							
**Diet for high blood pressure**			
Yes	1				1				1				1			
No	2.42	1.30	4.51	0.01	2.44	1.31	4.54	0.01	1.93	1.25	2.98	0.01	1.68	1.09	2.60	0.02
**Consumption of vegetables**			
Yes	1								1				1			
No	1.20	0.89	1.62	0.22					1.39	0.99	1.94	0.05	1.40	1.00	1.97	0.05
**Consumption of fruits**			
Yes	1								1				1			
No	1.25	0.94	1.67	0.13					1.80	1.32	2.46	0.01	1.81	1.33	2.47	0.01
**High contribution of UPF in the diet**			
No day	1								1							
One day	0.96	0.60	1.52	0.85					1.50	1.00	2.25	0.05	1.48	1.00	2.20	0.05
Two days	1.06	0.50	2.23	0.89					2.24	1.24	4.02	0.01	2.27	1.25	4.12	0.01

OR, Odds ratio; CI, Confidence interval; UPF, Ultra-processed food.

^a.^Underweight (IMC < 22 kg/m^2^), normal weight (22 kg/m^2^ ≥ IMC < 27 kg/m^2^), overweight (IMC ≥ 27 kg/m^2^).

^b^1st: < US$ 226; 2nd: ≥ US$ 226 to < US$ 391; 3r: ≥ US$ 391 to < US$ 678; 4th: ≥ US$ 678.

^c^Single person (household composed of a single person), couple (household composed of the head of household and spouse - without child), Mixed (other types of households composed in other ways than those above).

P-value less than 0.05 is considered significant.

Goodness of fit of the multiple model: Male: p = 0.99 and female: p = 0.29. These results indicate a good fit.

Table 3 presents the adjusted odds ratios by sex, derived from the final model, which retained only variables with at least one category statistically significant at the 5% level. Among men, two factors were significantly associated with adding salt to food at the table. Men who reported not following a diet for high blood pressure were more than twice as likely to add salt compared with those who were on such a diet (adjusted OR = 2.44, 95% CI: 1.13–4.54). In addition, men living alone had a 62% higher likelihood of adding salt compared with those living with others (adjusted OR = 1.62, 95% CI: 1.08–2.43). Among women, the final model identified significant associations with age, area of residence, household arrangements, reporting not following a diet for high blood pressure, vegetable consumption, fruit consumption and a high contribution of UPF in the diet. The adjusted OR indicates that women not following a diet for high blood pressure were 68% more likely to report adding salt to food at the table compared with those who adhered to such a diet (adjusted OR = 1.68, 95% CI: 1.09–2.60). Similarly, the odds were 40% higher among women who did not consume vegetables compared with those who did (adjusted OR = 1.40, 95% CI: 1.00–1.97), and 81% higher among those who did not consume fruit compared with those who did (adjusted OR = 1.81, 95% CI: 1.33–2.47). Additionally, women residing in urban areas were more than twice as likely to report this habit compared with those living in rural areas (adjusted OR = 2.44, 95% CI: 1.60–3.72) and women who had two days with a high contribution of UPF in their diet compared with those who had no days (adjusted OR = 2.27, 95% CI: 1.25–4.12).

## 4 Discussion

This study aimed to investigate the association between socio-demographic, anthropometric and dietary characteristics and the habit of adding salt to food at the table. We hypothesized that the habit of adding salt to food at the table would be associated with specific socio-demographic characteristics of older adults, with particular emphasis on gender, health conditions and urban-rural areas. We found that, among men, those who were on a diet for high blood pressure and living alone showed a significant association with the habit of adding salt to food at the table. In contrast, several characteristics were associated with this habit in women, such as not following a diet for high blood pressure, not eating vegetables, not eating fruit, having a high contribution of UPF in the diet, and living in urban areas, suggesting that the salt consumption patterns in women are influenced by a complex interplay of dietary habits and health awareness (41).

### 4.1 Gender differences in the habit of adding salt to food at the table

Regarding gender differences, our results demonstrate that men were more likely to add salt to foods at the table compared to women (12.7% vs. 9.4%, respectively). Similar patterns were also observed by Castro et al. (24), with a higher prevalence of salt added to prepared foods in men than in women (9.8% vs. 6.9%). Men in the study consumed more salt than women as estimated by 24-h urinary sodium (Na) excretion (11.7 v. 9.6 g salt/d; *P* < 0·0001) (42). Mill et al. (14), reinforced that gender plays a crucial role in dietary habits within the Brazilian context.

Gender differences in knowledge, attitudes, and behaviors related to salt intake have been identified as possible explanations for these findings (22, 43–45). Such differences may influence not only the prevalence of discretionary salt use but also broader dietary practices and health outcomes, reinforcing the importance of considering gender when developing targeted public health strategies.

For example, a study conducted in Germany highlighted that men are generally more likely to add salt to their food, probably due to cultural and social norms that influence food preferences and dietary habits, while women report reading the salt content on nutritional labels more frequently, use natural seasonings instead of salt, and avoid eating out (41). In addition, men tend to have less adherence to dietary guidelines, including salt reduction recommendations, which may explain their higher habit of adding salt to foods at the table (43).

In line with these findings, Devault (46) shows that food-related practices are strongly influenced by gender, with women more often taking on the roles of preparing healthy food and providing care within households. This gendered division of dietary responsibility may explain why women are more vigilant about salt use and nutritional information. Conversely, men tend to be less involved in these care routines, which could reinforce the behavioral differences observed in salt consumption and dietary adherence (46, 47).

### 4.2 Household arrangements and the habit of adding salt to food at the table

In the present study, older men living alone were more likely to add salt to food at the table compared to those who lived with others (adjusted OR = 1.62, 95% CI: 1.08–2.43). Previous research supports this association. Castro et al. (24) highlighted that living alone may negatively influence dietary habits, potentially leading to increased salt intake among older adults. Tani et al. (48) found that people living alone tended to have unhealthier diets, including higher salt intake, than people living with others, suggesting that the lack of social interaction during meals may lead to less attention to diet quality and an increased reliance on adding salt. This pattern is attributed to factors such as limited motivation or skills to cook, less attention to diet quality and greater reliance on ready-to-eat meals (49, 50).

On the other hand, older women living with a partner and without children were less likely to add salt to food at the table compared with those who lived alone (adjusted OR = 0.58, 95% CI: 0.40–0.85). A possible explanation for this finding is that cohabiting with a partner may encourage more consistent dietary patterns and reduce reliance on ultra-processed or away-from-home foods, which tend to be high in sodium (51).

### 4.3 Impact of high blood pressure on the habit of adding salt to food at the table

Our study found that that being on a diet for high blood pressure was inversely related to the habit of adding salt to food at the table among men, consistent with findings from other population-based studies (42, 52). This finding suggests bigger health awareness among men, particularly those managing hypertension (12, 53–55).

The Global Burden of Disease (GBD) study 2021 (8) reported better adherence to low-sodium diets among hypertensive patients in high-income countries, where public health campaigns and access to low-sodium alternatives are more widespread. These findings highlight the importance of strengthening health systems to support initiatives to provide targeted interventions, especially to improve dietary adherence and promote better health conditions among hypertensive adults.

### 4.4 Fruit and vegetable consumption and the habit of adding salt to food at the table

According to our findings, older women who eat fruit or vegetables were less likely to add salt to food at the table, which aligns with findings by Islam et al. (56). This result might be attributed to the inherent flavors present in vegetables and fruit, which diminish the need for additional salt to enhance taste. Public health organizations recommend diets rich in fruits and vegetables along with sodium reduction strategies, including limiting the addition of salt at the table (70).

Plessz and Guéguen (57) highlight gendered patterns in eating behaviors, showing that women tend to adopt healthier dietary practices more consistently than men. This includes consuming more vegetables and preparing food in a way that is more health-conscious. These findings suggest that these patterns are shaped not only by individual preferences, but also by social expectations and roles (57). This reinforces the idea that gender is an important dimension in nutritional behaviors and preventive health actions.

### 4.5 Impact of high contribution of UPF in the diet on the habit of adding salt to food at the table

A higher contribution of UPF (ultra-processed foods) to the diet was significantly associated with an increased likelihood of adding salt to food at the table. Among women, those with two days of high UPF intake had more than twice the odds of this behavior compared with those with no days (adjusted OR = 2.27, 95% CI: 1.25–4.12). Liem et al. (71) highlight that regular exposure to high-sodium foods can lead to a blunted perception of saltiness, leading individuals to develop a stronger preference for salty tastes and to seek additional salt in their diet. This preference is driven by sensory adaptation, in which repeated exposure to high sodium levels reduces sensitivity, prompting individuals to seek more salt to achieve the same perceived flavor intensity (32). As a result, women accustomed to ultra-processed foods may perceive home-cooked meals as bland and compensate by adding salt at the table.

Encouraging a gradual reduction in discretionary salt use, increasing awareness of hidden sodium in UPFs, promoting minimally processed foods and providing education on taste adaptation and sodium reduction may help to mitigate the health risks associated with excessive sodium intake (58, 59). Furthermore, public health strategies aimed at reducing discretionary salt use in women should focus on promoting minimally processed foods and providing education on taste adaptation and sodium reduction (70).

### 4.6 Impact of high contribution of UPF in the diet on the habit of adding salt to food at the table

Concerning area of residence, women living in urban areas were more than twice as likely to add salt to their food compared to those living in rural areas. On the contrary, Zhang et al. (60) found that rural residents in China had lower knowledge and weaker practices related to salt reduction, likely reflecting disparities in health resources and access to education. Higher levels of health literacy are often related to a better understanding of the risks associated with excessive salt intake (2).

According to the literature, the urbanization process in many regions of the world has also led to differences in exposure to UPF, generally higher in salt content (61). Conversely, rural populations tend to consume traditional diets, usually based on “*in natura*” foods, which may involve less discretionary salt use in their preparations (8, 62). Based on these findings, dietary and health promotion approaches, individually or in community-based programs in urban areas, should not only discourage the habit of adding salt at the table but also reinforce the importance of reducing ultra-processed foods.

### 4.7 Limitations and strengths

The study has some limitations. First, its cross-sectional design precludes causal inference, allowing only the identification of associations. Second, reliance on self-reported dietary habits, including salt addition, may have introduced social desirability bias, with participants potentially underreporting behaviors perceived as unhealthy (63). Third, not all food sources containing or rich in sodium were captured from the datasets used, which may influence the accuracy of estimated sodium availability and intake patterns. Finally, the use of data from the 2017–2018 NDS may limit the current relevance of the findings due to changes in dietary patterns since the data collection period.

Despite its limitations, this study has important strengths. The use of a nationally representative sample enhances the generalizability of the findings to the Brazilian population. Furthermore, the stratification of the analysis by gender provides valuable comprehension of the differential impacts of these factors on men and women, contributing to a better understanding of salt habits. The study also makes a significant contribution to public health by identifying key target areas for potential interventions aimed at reducing discretionary salt use in older adults.

### 4.8 Future directions

International studies have shown that while global guidelines, such as those issued by the **(author?)** (70), provide a valuable framework for reducing sodium intake, local adaptations are crucial to promote healthier dietary habits. For example, acknowledging possible limitations or barriers to progress in salt reduction within a context is vital for successful and effective consumer awareness campaigns, as suggested by prior studies (64–67). In a scoping review, Endaltseva et al. (68) identified that although many individuals are aware of the risks associated with excessive salt intake, this knowledge does not necessarily translate into reduced consumption. The authors emphasize the discrepancy between knowledge, attitudes, and behaviors, highlighting the need for comprehensive, multisectoral policies to encourage adherence to salt reduction recommendations. Complementing this perspective, Endaltseva and Dupuy (69) show that decisions regarding salt use are not only nutritional but also embedded in social meanings and ethics of care, suggesting that cultural, relational, and moral dimensions influence how individuals manage salt in everyday cooking, which has implications for how public health guidelines are interpreted and enacted in practice.

Therefore, this study provides substantial insights into gender, health conditions, fruit and vegetable intake and urban residency related to over consumption of salt, particularly among Brazilian older adults. Given the aging population and the high prevalence of hypertension and cardiovascular diseases in the Brazilian population (21), targeted interventions are critical. Public health campaigns should focus on raising awareness about the risks of excessive salt intake and promoting healthier alternatives, such as the use of herbs and spices for flavoring food. Additionally, efforts to increase fruit and vegetable consumption and reduce ultra-processed foods consumption could help mitigate the impact of high sodium intake on health.

## 5 Conclusion

This study underscores gender-specific differences, with men more likely to add salt to foods at the table, compared to women. Furthermore, key predictors of this behavior were also identified, such as not following a diet for high blood pressure in both sexes, and not consuming fruit and vegetables, living in an urban area and having a high contribution of UPF in the diet in women. These findings reinforce the need for continuous monitoring and tailored health promotion activities that address the underlying practices influencing excessive salt intake among Brazilian older adults.

## Data Availability

The raw data supporting the conclusions of this article will be made available by the authors, without undue reservation.
